# Leisure Participation of Taiwanese Families Raising Children with Developmental Delays and Disabilities

**DOI:** 10.3390/children12101326

**Published:** 2025-10-02

**Authors:** Ya-Jung Lin

**Affiliations:** Department of Early Childhood Education, National Taichung University of Education, Taichung 403, Taiwan; yajung@mail.ntcu.edu.tw

**Keywords:** leisure participation, developmental delays, disability, health literacy, inclusive recreation

## Abstract

**Highlights:**

**What are the main findings?**

- Neighborhood parks were central to families’ leisure participation, but access was constrained by uneven distribution, finances, and social attitudes.
- Both personal and organizational health literacy shaped how families navigated barriers and created leisure opportunities.

**What is the implication of the main finding?**

- Families sometimes understood leisure as supporting children’s developmental needs and readiness for school, not only as recreation.
- Strengthening inclusive infrastructure and coordinated community services can reduce inequities and foster participation.

**Abstract:**

Background/Objectives: Leisure participation is vital for children’s development and family inclusion, yet families of children with developmental delays and disabilities face significant barriers. Guided by a health literacy framework, this study examined how personal and organizational health literacy shape access to inclusive leisure opportunities. Methods: Semi-structured interviews were conducted with 14 caregivers of young children (aged 2 to 6 years) with developmental delays and disabilities. A qualitative content analysis was applied to identify family and environmental factors shaping leisure participation. Results: Families with stronger personal health literacy engaged in diverse leisure activities, prioritizing children’s development through park visits and structured home routines. In contrast, weak organizational health literacy—reflected in limited inclusive facilities and support systems—restricted opportunities, increased caregiver stress, and forced adaptations such as traveling farther or rescheduling activities. These barriers underscored families’ vulnerability to exclusion while also highlighting their resilience in navigating daily life. Conclusions: This study demonstrates that health literacy functions at both personal and organizational levels to shape leisure participation. Beyond identifying barriers, it shows that leisure is intertwined with developmental needs and school readiness. By applying a health literacy lens, the study contributes to understanding family dynamics in inclusive leisure and underscores the need for responsive community services and inclusive policies.

## 1. Introduction

Families raising young children with developmental delays or disabilities face unique challenges in supporting their children’s development. Within this context, leisure activities are essential contexts for children’s social, emotional, and cognitive development, particularly for those with disabilities who often face barriers to participation. These activities enhance children’s abilities in language acquisition, problem-solving, social competence, and self-regulation [1]. Yet studies show that the participation of preschoolers with disabilities is shaped less by children’s own initiation and more by family choices and available opportunities [2,3]. This suggests that while leisure provides meaningful opportunities for early learning and inclusion, the extent of participation is closely determined by family and environmental factors [2,3], making leisure a critical marker of inclusion and social connectedness for families raising children with disabilities.

International research further identifies barriers and facilitators across three dimensions: children, family, and environment. Children often face developmental challenges that limit their participation [4,5,6], while family barriers relate to attitudes within the nuclear family and their social circles [4]. Environmental barriers include cultural stigma and a lack of supportive infrastructure [7]. Conversely, maternal involvement, family support, and connections with other families can help overcome these obstacles [5,7]. Leisure participation is also influenced by children’s gender, age, and family economic status [8]. A systematic review highlights that the influence of family economy has been reported inconsistently across studies; instead, process-related aspects such as family health and well-being, parental attitudes, behaviors, beliefs, and available resources may play an equally critical role [9]. In many Asian families, structured activities such as music lessons or academic tutoring are prioritized to meet cultural norms of achievement, sometimes at the expense of child-led play [2]. Ensuring that leisure is child-centered rather than driven by societal expectations is therefore critical for inclusive participation [10].

In Taiwan, when young children are diagnosed with developmental delays or disabilities, families are connected to early intervention services through community-based child development case management centers. Social workers at these centers act as local coordinators, linking families with individualized services that include individualized education programs, therapeutic intervention, and family support services [11,12,13]. However, these formal interventions largely occur in clinical or educational settings and do not directly address how families engage in everyday community leisure. Families raising children under six with developmental delays or disabilities face particular challenges in this regard.

At the policy level, Taiwan incorporated the principles of the United Nations’ 1989 Convention on the Rights of the Child (CRC) and the 2006 Convention on the Rights of Persons with Disabilities (CRPD) into its 2014 Implementation Acts. These led to initiatives such as the Taipei City Government’s Play for All program, which since 2016 has promoted the design of inclusive playgrounds and public spaces [14,15]. These inclusive playgrounds incorporate features such as barrier-free equipment, multi-sensory play zones, and varied play structures that enable children with and without disabilities to engage side by side. Beyond recreation, they serve as important contexts for parent–child interaction and peer learning, supporting early communication, social competence, and inclusion. Yet despite these commendable efforts, challenges persist. The 2022 Concluding Observations from the International Review Committee of the CRC and CRPD highlighted deficiencies in Taiwan’s playgrounds and public spaces, calling for further improvements [16,17].

Cultural factors, such as entrenched expectations that mothers should serve as the primary caregivers and the stigma associated with disability, further intensify family isolation [18,19]. These pressures intersect with accessibility barriers in the community, including uneven provision of barrier-free playgrounds, limited transportation options, and fragmented information about suitable activities. As a result, families often struggle to translate individualized developmental supports into inclusive leisure opportunities. These challenges reflect cultural, structural, and policy-related dimensions, as also noted in the CRC/CRPD Concluding Observations, which highlighted deficiencies in inclusive facilities and the need for stronger governmental coordination.

Health literacy is essential for families to effectively access and utilize resources related to their children’s leisure activities [20,21], and it provides a useful lens to analyze these dynamics. It is broadly defined as people’s knowledge, motivation, and competencies to access, understand, appraise, and apply health information [22]. According to Healthy People 2030, personal health literacy refers to an individual’s ability to seek, comprehend, and apply information, while organizational health literacy reflects the extent to which institutions communicate information clearly and design inclusive systems that support decision-making [23]. Building on this, Barch et al. [24] highlight ten attributes of health literate organizations, such as simplifying access, integrating cultural and linguistic appropriateness, and ensuring equity, which are critical for enabling families to make informed choices about leisure and participation opportunities.

In this study, these two dimensions are analytically distinguished in line with these conceptualizations: personal health literacy refers to caregivers’ strategies for seeking, interpreting, and applying information about leisure opportunities, whereas organizational health literacy reflects how institutions and community systems ensure accessible facilities, inclusive services, and supportive communication. Their detailed application is elaborated in the Methods Section 2.1.

In the context of families raising children with developmental delays and disabilities, these two dimensions interact: parents’ capacity to locate and evaluate information depends heavily on whether organizations provide user-friendly, inclusive, and supportive structures. Despite growing international evidence, gaps remain in understanding how personal and organizational health literacy jointly shape leisure participation for families with young children with disabilities, particularly in non-Western contexts such as Taiwan. Unlike previous studies that categorized barriers and facilitators into child, family, and environmental domains, this study extends the discussion by applying a health literacy framework to reveal how families’ information-seeking abilities and organizational responsiveness interact to shape leisure participation. While local parks emerged as a central focus, the study also considers other leisure opportunities and the broader environmental and family factors influencing participation. This study aims to explore how family and environmental factors influence leisure participation among Taiwanese families raising children under six with developmental delays or disabilities, guided by the health literacy framework.

## 2. Materials and Methods

### 2.1. A Conceptual Framework

**Figure 1:**
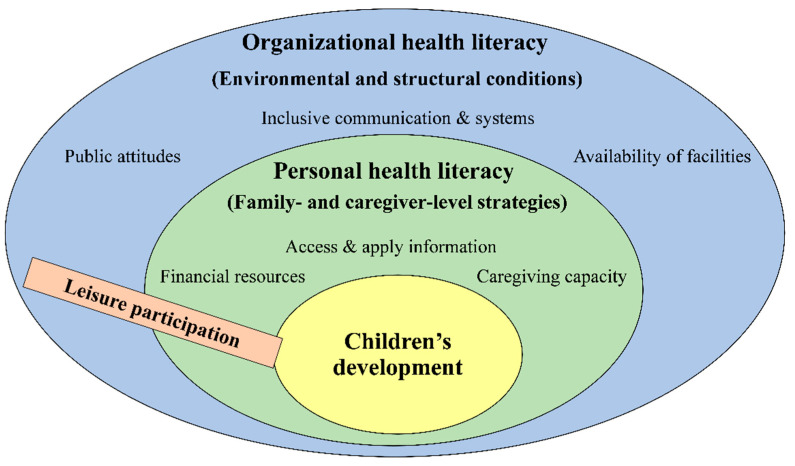
Framework of personal and organizational health literacy in leisure participation.

This study adopted a health literacy framework that conceptualizes leisure participation as shaped by the dynamic interplay between personal health literacy and organizational health literacy. Personal health literacy refers to caregivers’ ability to seek, interpret, and apply information about leisure opportunities, whereas organizational health literacy reflects the extent to which institutions ensure accessible facilities, inclusive services, and supportive communication. While these two dimensions are interdependent, they remain analytically distinct in this study: personal health literacy captures family- and caregiver-level strategies, whereas organizational health literacy captures environmental and structural conditions. Family-level factors (e.g., financial resources, caregiving capacity) and environmental conditions (e.g., public attitudes, availability of facilities) operate through these dimensions. This framework guided the analytic process, and Figure 1 presents the conceptual model, positioning leisure participation as a cross-cutting process that links family and environmental dimensions to children’s development.

### 2.2. Study Setting and Participants

This study was conducted in Changhua County, central Taiwan, where most townships have at least one public park. Parks are especially relevant in this context, as they represent the primary and most common settings where families with young children engage in everyday leisure activities. This context underscores the importance of examining how families navigate leisure opportunities and barriers in community spaces.

A total of 14 caregivers (12 mothers, 2 grandmothers) of children aged six or below with developmental delays or disabilities participated in the study (see Appendix A for participant characteristics). In this study, the term “caregiver” refers to parents or grandparents who were the primary caregivers of children with developmental challenges. Eligible families were those residing in the township for at least one year and with a formal diagnosis provided by medical clinics. Caregivers were recruited through convenience sampling, primarily via invitations from social workers in community-based child development case management centers, which serve as local coordinators of Taiwan’s early intervention system, and, in some areas, preschool teachers. Recruitment occurred immediately after the COVID-19 pandemic, when access to service sites was restricted and families’ willingness to participate was reduced. To accommodate caregivers’ availability and comfort, interviews were arranged in accessible public spaces such as convenience stores.

### 2.3. Data Collection Procedures

The study received ethical approval (NCUEREC-111-026) prior to recruitment. Preliminary interviews with service providers were conducted to refine the interview guide and ensure contextual relevance. The semi-structured guide drew on prior literature and the health literacy framework, with two domains: families’ experiences of leisure activities with children and perceived facilitators and barriers to participation, each containing 3–4 open-ended questions. All interviews were conducted by the author, who has professional training in early intervention and qualitative research. Interviews lasted 60–90 min and were arranged in accessible public spaces due to limited access to service sites in the aftermath of the COVID-19 pandemic. Informed consent was obtained through signed forms, and caregivers received NT$1600 as compensation. To ensure rigor, the researcher employed active listening, neutral probing, and follow-up questions to facilitate authentic narratives. All interviews were audio-recorded, transcribed verbatim, and anonymized to protect confidentiality.

### 2.4. Data Analysis

Data were analyzed thematically through a systematic coding process. Transcripts were reviewed line by line, and meaningful text segments were assigned initial codes. Codes were iteratively compared and refined across caregivers, then organized into matrices to identify recurring patterns and higher-order categories. Throughout the analysis, the health literacy framework functioned as a sensitizing lens, drawing attention to how personal (e.g., caregivers’ information use and family finances) and organizational (e.g., public attitudes and facilities) dimensions interacted with family and environmental factors in shaping leisure participation.

Although coding was conducted primarily by the author, reliability was enhanced through repeated cross-checking of codes and themes, as well as discussions with a research assistant trained in qualitative methods. Formal interrater reliability statistics were not calculated, but analytic rigor was supported by these validation strategies. To further strengthen rigor, data triangulation was employed by integrating interview transcripts with field notes documenting caregivers’ non-verbal expressions, interview settings, and observed social dynamics. This integration helped validate interpretations across multiple sources and distinguish explicit caregiver accounts from contextual nuances, thereby enhancing trustworthiness.

Finally, themes were organized within the health literacy framework, which structured both the presentation of results and the subsequent discussion. A deductive approach was used to compare emerging themes with existing literature while also highlighting unexpected findings. The analysis considered families’ accounts across different leisure contexts, including indoor facilities, community spaces, and neighborhood parks, to capture the range of participation experiences.

## 3. Results

### 3.1. Families’ Leisure Participation in Parks and Beyond

#### 3.1.1. Accessibility and Integration of Park Spaces

Neighborhood parks emerged as the most accessible and frequently used leisure spaces. Nine out of thirteen participants emphasized the importance of family recreation. For most families, proximity made parks convenient for daily use. In contrast, a few families (such as P02 and P06) reported fewer nearby options, requiring longer travel times or car trips (see Appendix B for detailed distribution of distances, travel times, and parks located within 500 m of participants’ homes).

Parks’ accessibility enabled their integration into families’ daily routines. Participants often described park visits as spontaneous, noting that these outings provided opportunities for children’s play and moments of respite for caregivers. As P09 shared: “It is just a five-minute walk to the park… whenever I feel tired, I say, ‘Let’s go outside to play.’” In contrast, families like P03 and P06 found regular visits more difficult due to the longer travel times required. As P03 reported, “There are no parks near my home, and it’s hard to take my three kids out, especially while I’m pregnant.” These accounts highlight how uneven park distribution limited some families’ ability to visit regularly.

Park visits also facilitated shared caregiving. As P01 noted: “My husband did not like taking our daughter out, but after some convincing, he would take her to the park to play, which gave me time to rest.” Such examples showed that proximity and ease of access allowed parks to serve as central spaces in families’ everyday caregiving practices.

#### 3.1.2. Participation in Indoor and Community-Based Facilities

Beyond neighborhood parks, families occasionally accessed other leisure resources, such as larger parks in neighboring townships or indoor facilities like parent–child centers. These spaces were typically used as supplements rather than being central to daily life. Larger parks were often described as “special outings,” offering broader green areas and novel play environments but requiring longer travel times. Parent–child centers provided safe indoor settings and structured programs, such as group games, storytelling, and guided activities, that parents valued, though limited availability and commuting demands restricted regular use.

Overall, indoor and community-based facilities enriched families’ leisure experiences by offering alternative play environments and structured programs. Yet their use remained occasional and opportunistic, unlike the routine integration of nearby parks. The challenges and adaptations related to these facilities are explored further in Section 3.2.

### 3.2. Factors Influencing Leisure Participation

Participants identified key factors shaping their leisure participation, including social attitudes, inclusiveness of leisure spaces, family finances, and parents’ information-seeking skills, all described in relation to their children’s developmental challenges.

#### 3.2.1. Social Exclusion in Public Playgrounds

Participants widely acknowledged parks as essential for children’s development, emphasizing that diverse play equipment—such as climbing structures, swings, and interactive installations—fostered physical strength, social skills, and cognitive growth while enabling peer interaction and independence.

Despite this supportive potential, some parents encountered unwelcoming attitudes from other parents, prompting them to switch playgrounds. For example, P07 initially traveled only 1.6 km (about five minutes by motorcycle) to a nearby park, but after experiencing unfriendly interactions, she began commuting twice the distance and time to a different playground. Participants linked these experiences to broader societal biases in Taiwan, noting that children’s developmental issues were often attributed to maternal factors. As one mother explained: “People always ask me what I did wrong during pregnancy, as if my child’s condition is my fault.” (P12) Such biases generate stigma and render public spaces less inclusive. For instance, many playgrounds lack sensory-friendly equipment, clear behavioral guidelines, or designated areas where children with developmental challenges can safely interact with peers.

Some participants also reported avoiding outdoor activities altogether, citing the emotional burden of managing strangers’ reactions to their children’s disabilities. Their accounts illustrate how stigma and negative social judgments directly affected leisure choices:

“It is hard to take my child out because of her condition, and others’ impolite reactions make me feel guilty. I would rather stay home.” (P04)

“I struggle to manage my child’s aggressive behavior, and criticism from others makes us prefer to stay at home.” (P10)

These accounts described how negative social attitudes, combined with the lack of inclusive design, discouraged families from using public parks.

#### 3.2.2. Challenges and Needs in Recreational Spaces

Some participants noted that their children, particularly those with severe developmental challenges, struggled to navigate conventional playground equipment. These challenges prompted families to seek alternative spaces such as parent–child centers. Participants valued these settings for their safer indoor environments, staff supervision, and age-appropriate equipment. As P01 mentioned:

“We can get more care to keep safety, and the courses there are fruitful for us to learn how to play with our child.” (P01)

Structured activities—such as craft making, music sessions, or circle-time games—were also easier for children with developmental challenges to follow. However, participants noted that group activities could be difficult when rules were unclear or inflexible, which sometimes led to unintentional exclusion. As P05 explained: “It’s difficult for my child to understand the activity rules, and he needs more waiting time. But typical children rarely accommodate him to join the activities.” She further proposed, “Is it possible to set aside time? I have thought about it—there are seven days in a week. Could two days be reserved for these special children?”

Beyond this proposal, P05′s reflections also revealed how parents frequently distinguished between “typical” and “special” children. When further asked about her expectations for inclusion, she acknowledged, “It is what I hope for, though parents of typical children may feel differently.” In the absence of such arrangements, some families turned to facilities farther from home.

#### 3.2.3. Financial Constraints in Leisure Choices

Family finances significantly shaped leisure choices, with participants falling into two broad groups: those facing economic constraints and those with greater financial stability. For families with limited resources, options were typically constrained to nearby parks. P13, a single mother, explained that the burden of balancing work and medical expenses for her child’s therapies, limiting them to a local park within 0.9 km:

“Due to financial constraints, I cannot afford to take my child beyond our township for recreation. My child still needs therapy services, so we make the most of the nearby park. I am cautious with every penny.” (P13)

By contrast, financially stable families enjoyed more flexibility and could occasionally travel further for leisure, enhancing family time and supporting their children’s development. P09 and P12, for example, described how they traveled between 9.3 and 14.3 km to parks in other townships on weekends, noting that these trips offered more varied activities and opportunities for parent–child connection:

“There is a park in a nearby county with a long side and a grassy area where kids fly kites. My husband and I enjoy helping our son make new friends there.” (P09)

“These parks offer activities that support my child’s motor skills and provide my husband with time to connect with our child.” (P12)

These accounts showed that families with greater financial stability engaged in longer-distance leisure trips, whereas those with financial constraints relied more on nearby, low-cost options.

#### 3.2.4. Navigating Leisure Opportunities for Child Development

Some parents described leisure not only as recreation or family bonding, but also as a way to prepare their children for school readiness. They noted that playground activities—such as climbing, waiting for turns, or group play—helped their children practice skills needed for preschool participation. P06, for example, explained that finding local park activities through Facebook gave her child early opportunities for peer interaction before entering school:

“Most of the activities I find on Facebook are in parks, giving my child chances to play with peers before preschool.” (P06)

Similarly, P07 described joining a Line learning group where weekly, parent-led park activities gave his son chances to practice social interactions with other children:

“I join a Line learning group where weekly, parent-led park activities help my son practice social interaction.” (P07)

Some participants also arranged home-based activities using online resources, particularly during the COVID-19 pandemic when in-person gatherings were restricted. P03, for example, explained how this approach supported her caregiving efforts:

“I often rely on activities from social media to ease caregiving stress, especially during the pandemic and my pregnancy, using YouTube videos to teach colors and counting.” (P03)

Parents described using digital platforms to locate park-based group activities or online educational videos, which they felt helped their children practice social skills and eased caregiving stress.

## 4. Discussion

This study examined how family and environmental factors influenced leisure participation among families raising children with developmental challenges. By situating the findings within the health literacy framework, the study extends current knowledge on how organizational and personal health literacy shape everyday experiences [23]. The results demonstrate that parks function not only as recreational settings but also as central infrastructures for family well-being and school readiness preparation. Moreover, the study highlights how families mobilize resources, negotiate barriers, and apply health literacy in practice to maintain their children’s developmental opportunities.

### 4.1. Organizational Health Literacy

#### 4.1.1. Parks as Central Infrastructures and Routine Leisure Spaces for Families

Findings highlight the centrality of parks as routine leisure spaces. While access varied, most families relied on nearby parks for children’s play and caregivers’ respite, underscoring their role in daily life. This aligns with research on organizational health literacy, showing that equitable distribution of community resources shapes participation [4,5]. Families’ persistence in integrating parks into daily routines—even when access was limited—demonstrates how organizational gaps force families to exercise personal health literacy as a compensatory strategy.

#### 4.1.2. Social Attitudes and Experiences of Exclusion

Families also described how stigma and negative attitudes discouraged leisure participation, echoing prior research on unwelcoming environments [7]. From an organizational health literacy perspective, inclusivity requires not only physical infrastructure but also social infrastructure—such as awareness campaigns and caregiver education—to foster understanding of developmental differences. Caregivers’ coping strategies, such as adjusting schedules or avoiding certain environments, illustrate how personal health literacy was mobilized in response to organizational and social shortcomings, further emphasizing the need for systemic change.

#### 4.1.3. The Need for Adaptive Recreational Spaces

Conventional park environments often failed to accommodate children with developmental challenges, highlighting families’ need for more adaptive and inclusive recreational spaces. Parent–child centers were valued for their safer settings and professional support, but remained limited in number and accessibility. From an organizational health literacy perspective, inclusive strategies could involve sensory-friendly hours, universal design in playgrounds, and the expansion of indoor facilities.

At the same time, caregivers’ suggestions for designated time slots reveal a tension: while tailored arrangements may reduce stress, they also risk reinforcing segregation if not accompanied by broader inclusion efforts. These family perspectives underscore how personal health literacy responses arise in the face of organizational gaps, showing that only when organizational design and family strategies align can environments be both accessible and equitable.

Above all, these findings highlight that organizational health literacy shapes leisure participation not only through the physical distribution of facilities, but also through the inclusivity of social attitudes and the adaptability of recreational environments. These organizational conditions define the structural boundaries of engagement. Families’ personal health literacy, while secondary, emerges in response to these boundaries and will be examined in the following section.

### 4.2. Personal Health Literacy

#### 4.2.1. Financial Constraints and Health Literacy Capital

Family finances strongly shaped leisure participation, with constrained families relying mainly on nearby parks while financially stable families accessed more varied opportunities. This finding extends prior evidence, which has inconsistently linked family economy to children’s participation, by showing how parental attitudes and available resources also matter [9].

Policy efforts to reduce transport costs and expand recreational facilities could help mitigate financial disparities. Importantly, our findings highlight families’ health literacy capital—their ability to mobilize information and strategies to sustain developmental routines. Even when financially constrained, some caregivers creatively adapted local parks, demonstrating how personal health literacy mitigated structural barriers and supported children’s developmental gains in social interaction, independence, and school readiness.

#### 4.2.2. Information-Seeking and Digital Strategies

Caregivers demonstrated personal health literacy through active information-seeking, often using digital platforms to locate activities, arrange interactions, or ease caregiving stress. This finding extends prior research linking personal health literacy with the capacity to seek, understand, and apply information [25,26].

Yet the reliance on self-directed searches revealed gaps in organizational health literacy. In contrast to contexts such as England, where coordinated digital systems support families [27], Taiwanese caregivers encountered fragmented resources spread across social media channels. These findings highlight the need for integrated, accessible platforms that centralize reliable information and link professional expertise with caregiver needs. Strengthening digital health literacy and developing coordinated information-sharing systems would reduce inequalities and embed leisure opportunities more effectively within developmental support.

Together, these findings highlight how personal health literacy enables families to navigate financial barriers and fragmented information systems by mobilizing strategies, resources, and digital tools. These capacities not only mitigate structural constraints but also position leisure as a pathway to children’s developmental gains in social skills, independence, and school readiness.

### 4.3. Synthesis and Implications

Our analysis shows how organizational and personal health literacy intersect to shape leisure participation among families raising children with developmental challenges. Uneven facility distribution, fragmented information, and negative social attitudes constrained access, while families responded by mobilizing financial strategies, digital resources, and developmental routines. This dual dynamic underscores both family resilience and systemic barriers.

The study makes two novel contributions. First, it reframes everyday leisure spaces—especially parks—not only as venues for recreation or bonding but as arenas for preparing children for preschool participation. This developmental perspective, rarely emphasized in previous research, highlights how leisure choices are embedded in school readiness and broader developmental trajectories. Second, the study extends the application of health literacy into the domain of family leisure, showing how both personal and organizational dimensions jointly influence participation. These contributions enrich the theoretical scope of health literacy while advancing understanding of family experiences.

For policymakers, these findings highlight several practical entry points. Local governments and municipal recreation bureaus could pilot sensory-friendly hours in public leisure spaces (especially parks), while urban planners integrate universal design principles into new playground construction. Health, education, and social welfare agencies could collaborate with community-based child development case management centers, which act as local coordinators of early intervention services, to establish integrated digital platforms. Such collaboration would reduce caregivers’ reliance on fragmented social media searches and ensure that developmental and leisure information is delivered through reliable, accessible channels. Internationally, Taiwan’s parent–child center model offers a transferable example of low-cost, community-based recreational infrastructure that combines developmental support with leisure opportunities. Conversely, experiences from countries such as England, where coordinated digital health platforms connect families and professionals, may inform efforts to strengthen organizational health literacy in Taiwan.

**Figure 2:**
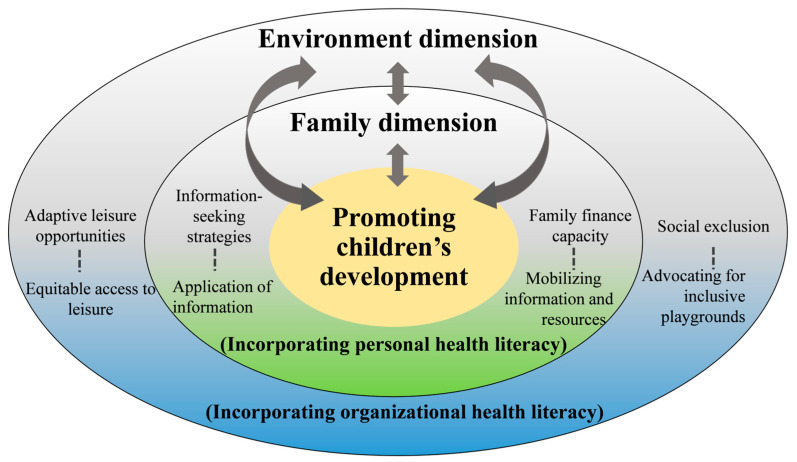
Conceptual framework of leisure participation shaped by family and environmental dimensions under the health literacy framework.

As summarized in Figure 2, the conceptual model highlights how family strategies (personal health literacy) and environmental conditions (organizational health literacy) interact dynamically. Leisure participation emerges as a cross-cutting process that both shapes and is shaped by family and community contexts, creating iterative cycles of developmental opportunities.

In sum, this study demonstrates how applying a health literacy lens to family leisure participation provides a deeper understanding of both systemic barriers and families’ adaptive strategies. By reframing leisure as part of children’s developmental preparation and extending the application of health literacy into everyday community contexts, the findings advance theoretical discussions on inclusion and family well-being. Future research could further test these insights across diverse populations and explore interventions that strengthen both personal and organizational health literacy.

## 5. Conclusions

This study emphasizes how families’ participation in leisure activities is influenced by both organizational and personal health literacy. Organizational factors, such as inclusive facilities and supportive public attitudes, interact with personal factors like family finances and caregivers’ ability to seek out information to shape children’s opportunities.

The study goes beyond simply documenting these dynamics; it provides new insights by illustrating that families often view leisure not just as recreation but as a means to support their children’s readiness for school. This perspective, which has not been emphasized in previous research, highlights the important developmental implications tied to leisure participation.

Enhancing caregivers’ health literacy is crucial for helping them navigate and utilize available resources, but these efforts cannot rely solely on families. Equally important is enhancing organizational health literacy—through inclusive facilities, positive public attitudes, and coordinated information systems—so that families encounter environments that actively reduce barriers and foster inclusion. Addressing these gaps requires coordinated efforts by local governments, service providers, and community organizations to create inclusive environments that reduce family stress and expand opportunities for participation.

This study has several limitations. The small sample of 14 caregivers from a single county limits generalizability, and the convenience sampling strategy—recruiting through service providers—may have underrepresented more isolated families. Most participants were mothers, so fathers’ perspectives were limited. As with many qualitative studies, reliance on self-reported interviews and the absence of a full double-coding process with interrater reliability may have constrained validity, despite efforts to enhance rigor through peer discussion and triangulation. Recruitment also occurred shortly after the COVID-19 pandemic, when lingering concerns reduced participation. Despite these constraints, the study provides timely insights into families’ leisure participation and health literacy. While these limitations should be noted, the study nevertheless provides timely insights into families’ leisure participation and health literacy. Future research could expand to more diverse samples, adopt longitudinal designs, include fathers as primary caregivers, and test interventions to strengthen health literacy and community inclusion.

## Data Availability

The data presented in this study are not publicly available due to confidentiality restrictions.

## Appendix A. Demographics of Participants

Of the 14 participants, 12 were mothers of children aged 2–6 years and 2 were grandmothers (P10 and P14) who served as the primary caregivers. Diagnoses among the children ranged from mild sensory impairments and mild to moderate intellectual disabilities to severe neuromusculoskeletal impairments, illustrating the diversity of developmental challenges represented in the sample.

**Table A1:** Demographic characteristics of participants.

Participant Code	Primary Caregiver Role	Child’s Age	Developmental Diagnosis
P01	Mother	6	cognitive delays
P02	Mother	3	cognitive and motor delays
P03	Mother	6	cognitive delays
P04	Mother	2	severe intellectual disability with neuromusculoskeletal impairments
P05	Mother	6	severe intellectual disability with neuromusculoskeletal impairments
P06	Mother	3	cognitive and motor delays
P07	Mother	5	moderate mental disability
P08	Mother	4	cognitive and speech delays
P09	Mother	3	minor sensory impairment of the ear structure
P10	Grandmother	5	social, emotional, and behavioral delays
P11	Mother	3	cognitive and speech delays
P12	Mother	5	mild intellectual disability
P13	Mother	5	mild intellectual disability
P14	Grandmother	4	cognitive and speech delays

## Appendix B. Geographical Distribution, Travel Distance, and Transportation to Parks

Appendix B provides information on park accessibility for each participant. In this study, a 500 m buffer was created around each participant’s residence using QGIS software to represent walkable access. This radius is commonly applied in public health and urban planning research as a proxy for walkable distance [28]. Only four participants had at least one park within this buffer. Nevertheless, most families reported using parks as their main leisure space, often traveling beyond their immediate neighborhoods. Transportation times were reported by participants during interviews, reflecting their perceived travel experiences. Distances were calculated using Google Maps from participants’ residences to the parks they most frequently visited. These complementary measures—perceived and objective accessibility—demonstrate how families were willing to travel beyond immediate walkable distances, underscoring the centrality of parks in their leisure routines. Detailed results are presented in Table A2.

**Table A2:** Travel distance and transportation modes of participating families.

Participant Code	No. of Parks Within 500 m Buffer	Calculated Distance (km)	Reported Travel Time (min)	Transportation Mode
P01	0	1.0	14 min	Walk
P02	0	9.6	20 min	Car
P03	0	NA	NA	NA
P04	1	NA	NA	NA
P05	NP	NP	NP	NP
P06	0	3.6	10 min	Car
P07	0	3.4	10 min	Motorcycle
P08	0	0.65	10 min	Walk
P09	1	0.29	5 min	Walk
P10	0	1.0	10 min	Motorcycle
P11	0	3.5	15 min	Motorcycle
P12	0	NP	NP	NP
P13	3	0.9	5 min	Motorcycle
P14	1	0.35	5 min	Walk

NA = Not available (no travel experience reported during interview, e.g., families restricted by parental pregnancy or child-related developmental constraints). NP = Not provided (residential address missing, distance calculation not possible).
